# UPLC-Q-Exactive Orbitrap-MS-Based Untargeted Lipidomic Analysis of Lipid Molecular Species in Spinal Cords from Different Domesticated Animals

**DOI:** 10.3390/foods12193634

**Published:** 2023-09-30

**Authors:** Na Li, Long Xu, Hongbo Li, Zhenbin Liu, Haizhen Mo, Yue Wu

**Affiliations:** 1 College of Food Science and Engineering, Central South University of Forestry and Technology, Changsha 410004, China; 20200100082@csuft.edu.cn; 2 School of Food Science and Engineering, Shaanxi University of Science and Technology, Xi’an 710021, China; hongbo715@163.com (H.L.); zhenbinliu@sust.edu.cn (Z.L.); mohz@sust.edu.cn (H.M.); 3 College of Food Science and Technology, Henan Agricultural University, Zhengzhou 450002, China; longxu@henau.edu.cn

**Keywords:** domesticated animals, spinal cords, fatty acid composition, lipidome, canonical correlation analysis, LC-MS

## Abstract

Lipids are crucial components for the maintenance oof normal structure and function in the nervous system. Elucidating the diversity of lipids in spinal cords may contribute to our understanding of neurodevelopment. This study comprehensively analyzed the fatty acid (FA) compositions and lipidomes of the spinal cords of eight domesticated animal species: pig, cattle, yak, goat, horse, donkey, camel, and sika deer. Gas chromatography–mass spectrometry (GC-MS) analysis revealed that saturated fatty acids (SFAs) and monounsaturated fatty acids (MUFAs) were the primary FAs in the spinal cords of these domesticated animals, accounting for 72.54–94.23% of total FAs. Notably, oleic acid, stearic acid and palmitic acid emerged as the most abundant FA species. Moreover, untargeted lipidomics by UPLC-Q-Exactive Orbitrap-MS demonstrated that five lipid classes, including glycerophospholipids (GPs), sphingolipids (SPs), glycerolipids (GLs), FAs and saccharolipids (SLs), were identified in the investigated spinal cords, with phosphatidylcholine (PC) being the most abundant among all identified lipid classes. Furthermore, canonical correlation analysis showed that PC, PE, TAG, HexCer-NS and SM were significantly associated with genome sequence data. These informative data provide insight into the structure and function of mammalian nervous tissues and represent a novel contribution to lipidomics.

## 1. Introduction

Domestic animals are a primary source of animal fat for human consumption. Despite the prevalent use of their adipose tissues, other anatomical components, such as viscera and spinal cords, often remain underutilized, contributing to considerable wastage [1]. While China and other countries have traditionally consumed spinal cord tissue, the comprehensive characterization of the associated lipids and other molecular constituents has largely been overlooked. Lipids, with their myriad structures and functions, are pivotal to the evolutionary biology of organisms [2]. Their diversity emanates from an intricate interplay of factors, such as diverse polar head groups, variability in aliphatic chain lengths, the number and orientation of double bonds and potential modifications of hydroxyl group(s), among other unique chemical attributes. As of today (18 March 2023), 48,135 unique lipid structures have been cataloged in the LIPID MAPS^®^ structure database (NIH, USA) [3]. Lipids are fundamental to various physiological processes, including membrane fluidity, cellular signaling and energy metabolism [4,5,6].

Intriguingly, lipid components constitute approximately 50% of the dry weight of spinal cords [7]. Given that the spinal cord acts as a pivotal neural conduit for transmitting signals between the peripheral nervous system and the brain, the role of lipids within this context is especially significant. Previous research has documented that lipids are indispensable biomolecules for neurodevelopment, particularly in the formation of myelin [8]. Furthermore, disruptions in lipid metabolism within spinal cords have been linked to a spectrum of neurological disorders [9,10]. Therefore, a detailed analysis of the lipid molecular species in spinal cords is paramount for advancing our understanding of their diverse roles in the nervous system.

Lipidomics has emerged as a powerful tool to comprehensively characterize the lipidome and its involvement in health and disease. Recent advancements in mass spectrometry-based lipidomics, encompassing shotgun lipidomics [11], targeted lipidomics [12], untargeted lipidomics [13] and pseudotargeted lipidomics [14], have revolutionized our ability to characterize cellular lipidomes globally. These methodologies have substantially contributed to our understanding of lipid biology and its critical roles in various physiological and pathological conditions. Despite these strides, limited research has been conducted to compare lipid molecular species in the spinal cords of various domesticated animals and to comprehensively investigate the impact of genetic variations on these lipid profiles.

In this study, we employed GC-MS as an initial analytical technique to characterize the total fatty acid (FA) compositions of lipid extracts from the spinal cords of eight different domesticated animal species. Subsequently, an untargeted lipidomic approach based on UPLC-Q-Exactive Orbitrap-MS was employed to elucidate the specific lipid molecular species present in spinal cord lipid extracts. Finally, canonical correlation analysis (CCA) was performed to analyze the correlation between the genomic sequence and lipidome in each species. By integrating multi-omics datasets, this study aimed to elucidate the genetic factors governing lipid metabolism and their potential implications for spinal cord physiology and pathology. Examining these genotype–phenotype relationships could provide important insights into the molecular underpinnings of spinal cord function that may be conserved across diverse domesticated species. Furthermore, the comparative lipidomics analysis of the spinal cord lipidome may uncover lipid pathways that represent novel targets for supporting spinal cord health. Overall, understanding the fatty acid profile can shed light on the health implications of such dietary practices, including potential benefits or risks associated with consuming spinal cords. Furthermore, studying the composition of spinal cord fatty acids could help researchers to investigate links between diet and neurological conditions.

## 2. Materials and Methods

### 2.1. Samples and Chemicals

Vertebral specimens were procured from eight distinct species of domesticated animals: pig, cattle, yak, goat, horse, donkey, camel and sika deer. These samples were sourced from licensed meat-processing facilities in various geographical regions across China. Spinal cords were collected and immediately preserved at −20 °C until further analysis. All chemicals and solvents used for lipid extraction and sample analysis were of analytical grade or higher purity. They were acquired from Merck KGaA (Darmstadt, Germany) or Sigma-Aldrich (St. Louis, MO, USA).

### 2.2. FA Composition Analysis

Total lipids were extracted from the spinal cords of eight species of domesticated animals by sonication at 30 °C for 20 min using CHCl_3_/MeOH/H_2_O (2:1:0.75, *v*/*v*/*v*) containing 0.005% BHT at a sample/solvent ratio of 1:15 (*w*/*v*). Subsequently, phase separation was carried out through a separating funnel, and the lower layer was carefully collected. Then, the residual organic solvent was removed by a stream of nitrogen. Each sample was extracted three times. After lipid extraction, fatty acid methyl esters (FAMEs) were synthesized by incubating the extracted lipids (20 μg) at 85°C for 2.5 h in the presence of 1.5 mL of 1% H_2_SO_4_/MeOH (*v*/*v*). Then, 1.0 mL of saturated NaCl solution in distilled water, 1 mL of deionized water and 2.0 mL of *n*-hexane were added to the cooled mixture, which was then allowed to stand at room temperature to develop phase separation. The hexane phase was then carefully transferred to autosampler vials for GC-MS analysis.

The FAMEs were analyzed using an Agilent 7000D triple quadrupole mass spectrometry (Agilent Technologies, Santa Clara, CA, USA) equipped with an Agilent DB-23 column (30 m × 250 μm ID, 0.25 μm film thickness). Helium was the carrier gas at a flow rate of 2.25 mL/min. All measurements were performed in full-scan mode (*m*/*z* 50–500) with the following oven program: the initial temperature of 130 °C was held for 1 min and then raised to 230 °C at a ramp of 5 °C/min. The final temperature of 230 °C was held for 5 min, resulting in a total run time of 26 min. Sample injections of 1 µL were performed in split mode (1:20) using an autosampler. The FAMEs were identified by comparing retention times to a known C10–C24 FAME standard mix [15].

### 2.3. Lipidomic Analysis

The lipids were extracted from the spinal cords of eight domesticated animals following the method described by Matyash et al. [16] with minor modifications. Briefly, each spinal cord sample (100 mg) was thoroughly vortex mixed in a glass centrifuge tube with a Teflon lined cap using pre-cooled methanol (0.75 mL). Subsequently, pre-cooled methyl-*tert*-butyl ether (MTBE, 2.5 mL) was added, and the mixture was incubated at room temperature with shaking for 1 h. Ultrapure water at a volume of 0.625 mL was added to the mixture to induce phase separation. The upper organic phase was collected after centrifugation at 1000× *g* for 10 min. The lower phase was re-extracted with MTBE/MeOH/H_2_O (10:3:2.5, *v*/*v*/*v*, 1 mL) to collect any remaining lipids. The collected organic phase was combined and evaporated under a nitrogen flow. The dried lipid extracts were resuspended in isopropanol (IPA, 100 μL) and stored at −20 °C before lipidomic analysis. To ensure data reliability and evaluate system stability, a pooled quality control (QC) sample consisting of equal aliquots from each sample was prepared. Lipidomic analysis was performed using a Thermo Vanquish UPLC system coupled with a Thermo Orbitrap Q Exactive HF-X mass spectrometer operating in the data-dependent acquisition (DDA) mode. Each sample (5 µL) was injected into an Accucore C30 column (2.1 mm × 150 mm, 2.6 μm ID) at a flow rate of 0.35 mL/min, with the column temperature set at 40 °C. The mobile phase consisted of solvent A (MeCN:H_2_O = 60:40, *v*/*v*) and solvent B (IPA:MeCN = 90:10, *v*/*v*) containing 0.1% HCOOH and 10 mM CH_3_COONH_4_. The samples were injected into the column and eluted using a gradient of solvent B. The gradient started at 30% B for the first 2 min, then increased to 43% B at 5 min, and further increased to 55% B at 5.1 min. The gradient continued to increase to 70% B at 11 min, then rapidly increased to 99% B at 16 min and held constant until 18 min. At 18.1 min, the gradient rapidly decreased to 30% B and held constant until the end of the run at 20 min. The Q-Exactive HF-X mass spectrometer was used in both positive and negative ion modes with the following conditions: sheath gas at 20 arb; sweep gas at 1 arb; auxiliary gas in positive mode at 5 and negative mode at 7; spray voltage at 3 kV; capillary temperature at 350 °C; heater temperature at 400 °C; S-lens RF at 50; scan range from *m*/*z* 114–1700; and automatic gain control target at e^6;^ stepped normalized collision energy in positive mode at 25 eV and 30 eV and negative mode at 20 eV, 24 eV and 28 eV. The acquired raw data were processed using Compound Discoverer software (Thermo Fisher Scientific, Waltham, MA, USA), version 3.1, which included filtering, peak-picking, alignment, and normalization steps. The main parameters were as follows: retention time tolerance of 0.2 min; actual mass tolerance of 5 ppm; signal intensity tolerance of 30%; signal/noise ratio of 3; and minimum intensity of 100,000. The lipid molecular species were identified based on accurate mass measurements and lipid fragmentation patterns using the LipidBlast and Lipid MAPS databases [17,18].

### 2.4. Canonical Correlation Analysis (CCA)

**Figure 1:**
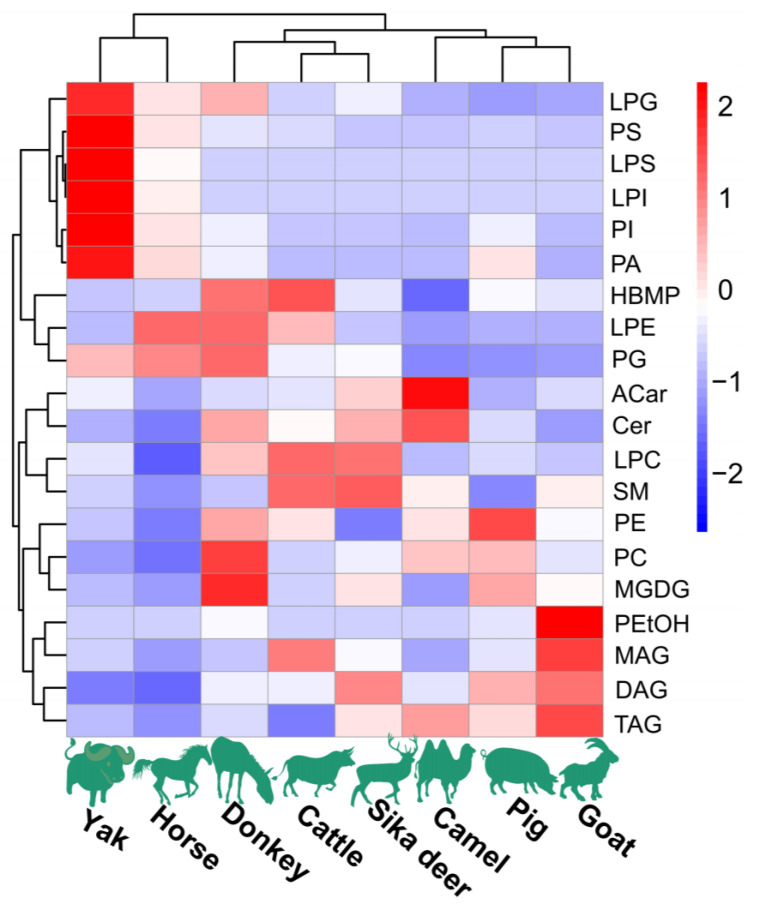
The content of major lipids in spinal cords of different species of domesticated animals. Each colored cell on the map corresponds to a peak area value of different lipid species. Red indicates high, and blue indicates low.

CCA is a well-established multivariate statistical algorithm used to explore the correlation between multivariate datasets and to identify the most relevant features [19,20]. This analytical method has been widely applied in pattern recognition and machine learning. CCA aims to find two weight vectors (*u* and *v*) that maximize the correlation coefficient between the linear combinations of two sets of random vectors (*X* and *Y*). Figure 1 illustrates the framework for correlation analysis of genomic sequence data and lipidomic data obtained from eight species of domesticated animals using the CCA model. The genomic sequence data were retrieved from NCBI, including NC_000845.1 (pig), HM118851.1 (donkey), DQ985076.1 (sika deer), MF925711.1 (cattle), NC_009628.2 (camel), KP273589.1 (goat), MF925712.1 (horse), NC_025563.1 (yak). $X\in {\mathbb{R}}^{n\times p}$ represents the genomic sequence data, and $Y\in {\mathbb{R}}^{n\times q}$ represents the lipidomic data, where *n* is the number of samples, and *p* and *q* are the characteristic dimensions of genomic sequence and lipidomic data, respectively. The correlation between the genome sequence and lipid profile data was analyzed using the CCA algorithm via MATLAB 2019b software to identify the canonical variables with the most significant correlations. Based on these canonical variables, features corresponding to genes and ROIs were extracted and analyzed to obtain the lipid profile variables with the highest correlation. Regularization was employed to reduce irrelevant features in X and Y to zero to mitigate issues with high dimensionality (genome sequence and lipid profile data) and small sample sizes (n). Consequently, sparse canonical variables and feature selection were achieved.

### 2.5. Statistical Analysis

Data were expressed as the mean ± standard deviation, with *p* < 0.05 considered statistically significant. The mean and standard deviation values were calculated using Microsoft Excel 2007 software. Principal co-ordinate analysis (PCoA) and hierarchical clustering analysis (HCA) were performed using R software (version R-3.4.3) [21].

## 3. Results

### 3.1. FA Profiles of Spinal Cords from Eight Species of Domesticated Animals

The compositions and contents of saturated and unsaturated (FAs) in the lipid extracts from spinal cords of eight species of domesticated animals are presented in Table 1. A total of 23 FAs were identified with high levels of SFAs [primarily palmitic acid (C16:0) and stearic acid (C18:0)] and MUFAs (mainly oleic acid (C18:1 n-9 *cis*)]. The total content of SFAs and MUFAs reached 74.64–97.75% of total FAs, with MUFAs comprising 37.60–58.41%. More specifically, palmitic acid and stearic acid accounted for more than 70% of all SFAs, while oleic acid accounted for approximately 50% of all MUFAs. Moreover, a higher level of PUFAs (25.36%) was identified in the lipid extracts from the spinal cords of horses compared to the other investigated species of domesticated animals.

### 3.2. Lipidomic Profiles of Spinal Cords from Eight Domesticated Animal Species

The lipidomic analysis of spinal cords from the eight investigated species of domesticated animals was conducted using UPLC-Q-Exactive Orbitrap-MS operated in positive and negative modes. Notably, the positive mode yielded a greater number of peaks in comparison to the negative mode, which demonstrated weaker responses. The determination of lipid molecular species was grounded in criteria including exact mass, retention time (RT), MS^2^ spectra and fragmentation pattern. Across these lipid extracts from domesticated animals, 22 lipid classes were identified, including PC, LPC, PE, LPE, PS, LPS, PI, LPI, PG, LPG, PA, HBMP, PEtOH, SM, Cer, Glucer, Shexcer, ACar, MGDG, MAG, DAG and TAG. The overall distribution of these lipids in the samples is shown in Figure 1. The analysis of individual lipid molecular species amounts within each category showed that PLs were the most abundant lipid class, comprising more than 50% of total lipids. Additionally, SLs and GLs were also substantially present in these spinal cord lipid extracts.

**Figure 2:**
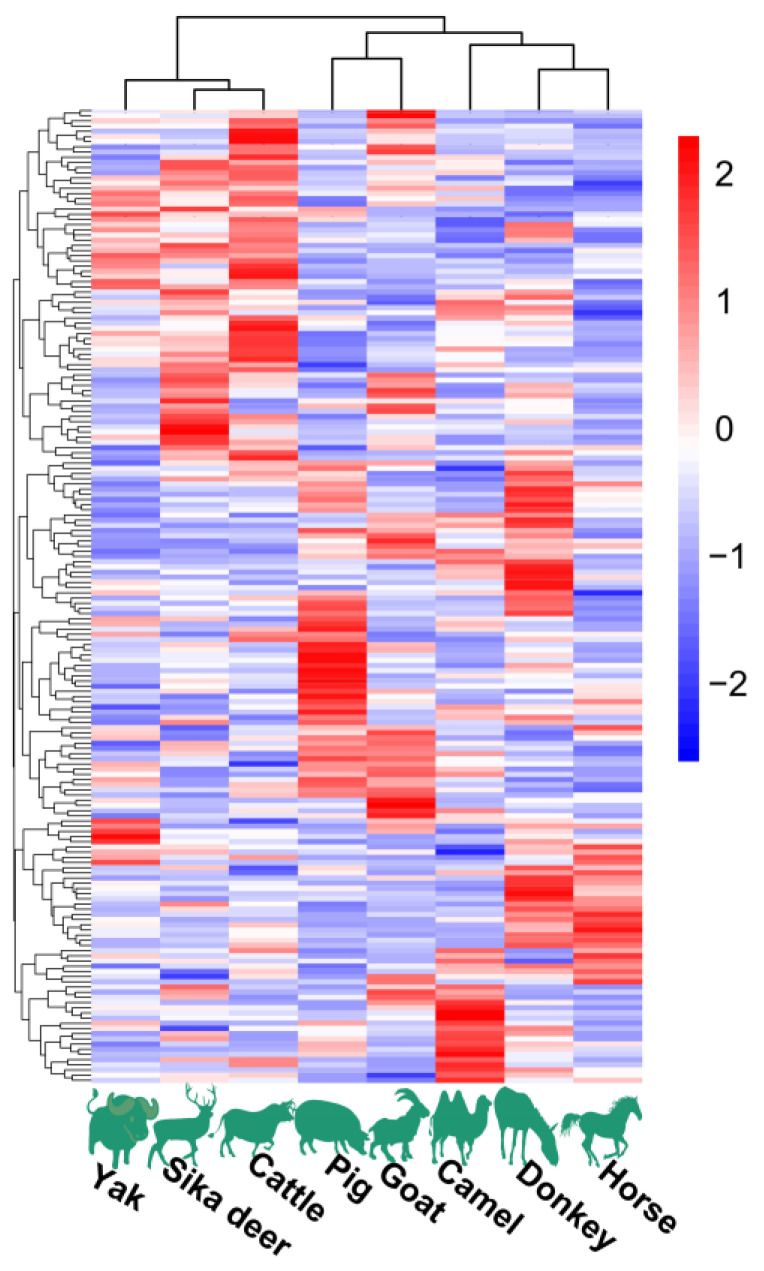
Lipid profiles in spinal cords of eight animal species.

**Figure 3:**
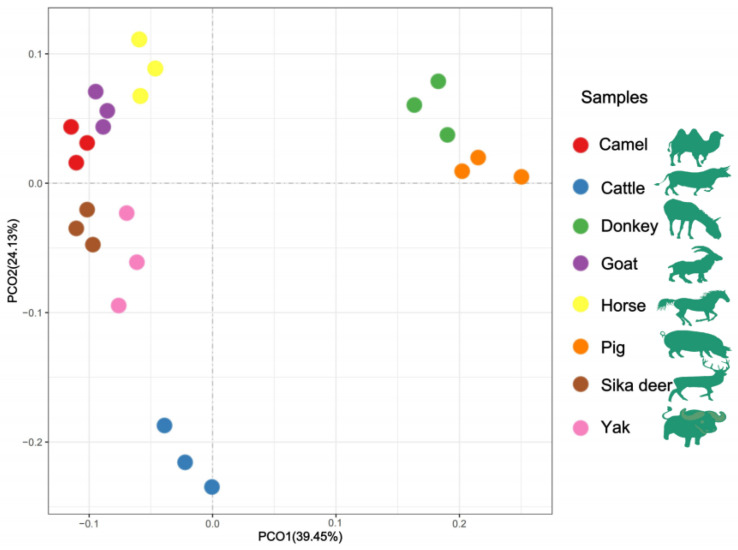
PCoA analysis of lipid molecules among the eight domesticated animals.

The HCA and PCoA results indicated that lipid molecules in the spinal cords of all eight animal species could distinguish between the species (Figure 2 and Figure 3). Figure 2 showed that the lipid profiles in the spinal cords of cattle and sika deer were similar. In addition, some similarity was also observed in the lipid profiles of the spinal cords between donkeys and horses. As illustrated in Figure 3, PCo1 and PCo2 accounted for 63.58% of the total variance in the dataset and demonstrated distinct sample separation into four groups: Group (1), camel, goat and pig; Group (2), donkey and horse; Group (3), sika deer and yak; and Group (4), cattle. The four sample groups occupied different quadrants, indicating that the lipid compositions of each group were distinct. Specifically, the lipid composition patterns in donkey and horse spinal cords were similar but markedly different from those in other animals. These results differ from those in Figure 2, possibly due to statistical differences in lipid data from different dimensions (Figure 2).

#### 3.2.1. PL Profiles

To compare the PL profiles across the spinal cords of eight distinct species of domesticated animals, the contents of PL molecular species within each class were determined based on their total peak areas (Figure 1). The primary constituents were identified as PC and PE, representing 96.7–99.3% of the total PLs. Notably, PC emerged as the predominant phospholipid class, contributing to more than 70% of the PL composition. While several other PL classes, including LPC, LPE, PS, PG, LPS, PI, LPI, LPG, PA, HBMP and PEtOH, were detected, their presence was merely in trace amounts. Delving deeper into the specifics, PC (16:0/18:1) was the most prevalent among the PC molecular species across all examined lipid extracts from spinal cords, followed in order by PC (18:0/18:1) and PC (16:0/16:1). Although a majority of the identified PCs were from the diacyl PC molecular species, certain alkyl-acyl PC species, such as PC (O-18:1/18:2), PC (O-20:0/18:3), PE (18:2e/18:1) and PG (18:1/18:1), were also recognized. When evaluating PE and PG species, PE (18:2e/18:1) and PG (18:1/18:1) stood out as the most abundant. A large proportion of alkenyl-PE, especially PE(P-18:0/18:2), was identified in the lipid extracts of spinal cords from donkeys, goats, horses, and yak. PS (18:0/18:1) was the predominant PS molecular species in the lipid extracts from the spinal cords of seven domesticated animal species, except for goat. PI (18:0/20:4) constituted the most significant proportion of PI molecular species in the lipid extracts from the spinal cords of seven domesticated animals, except for camel. Last, regarding lysoPE species, LPE (18:1) and LPE (20:1) were the most abundant molecular species.

#### 3.2.2. SL Profiles

The lipidomic analysis revealed that the SLs identified in the lipid extracts of spinal cords from the eight domesticated animal species accounted for 13.66–25.71%. The lipid extracts were categorized into four SL classes: SM, GluCer, Cer and ShexCer. Predominantly, the extracts exhibited large proportions of SM, ranging between 45.16% and 61.48%, followed by GluCer, which fluctuated between 28.82% and 34.24%. Notably, ShexCer was detected only in minimal, trace amounts In terms of specific molecular species, SM (d18:2/12:0) emerged as the dominant species in the lipid extracts derived from the spinal cords of pigs, sika deer, cattle, camels, goats and yaks. However, this particular SM (d18:2/12:0) manifested in lower quantities in donkey spinal cord extracts and was absent in those from horses. With regard to ceramide (Cer) species, both the Cer-NS and Cer-NDS species, composed of fatty acyls with chain lengths of 18–26, were identified in the lipid extracts from the spinal cords of eight species of domesticated animals. Cer-NS (d18:1/24:1) marked its prominence in the spinal cord lipid extracts from pigs, donkeys, sika deer, cattle, camels, goats and yaks. Conversely, the spinal cord lipid extracts from horses predominantly contained the Cer-NS (d18:1/24:0) species. Regarding GluCer, HexCer-NS and HexCer-NDS were identified as the major subclasses. Among these subclasses, HexCer-NS (d18:1/24:1) and HexCer-NDS (d18:0/24:1) were identified as the most abundant species for HexCer-NS and HexCer-NDS, respectively. Interestingly, only three SHexCer species, including SHexCer (d18:1/24:1), SHexCer (d18:0/24:1) and SHexCer (d18:1/25:1), were detectable in the lipid extracts derived from the spinal cords of eight domesticated animal species.

#### 3.2.3. GL Profiles

The lipidomic profiling revealed distinct glycerolipid (GL) compositions across the spinal cords of the eight domesticated animal species. The GL profile, including monoacylglycerol (MAG), diacylglycerol (DAG) and triacylglycerol (TAG), was determined only in positive mode. The results showed that the TAGs with fatty acyls of C12–C22 were the most abundant GL species in all of the animals’ spinal cord lipid extracts, accounting for 81.82–99.39%. Notably, TAG (16:0/18:1/18:1) was highly prevalent in the lipid extracts from the spinal cords of seven animal species but was not detected in cattle.

The DAG content differed significantly between species. Cattle spinal cord lipids contained the highest DAG level (17.70% of total GLs), while camels had relatively low DAG content (0.61% of total GLs). Specifically, DAG (16:1(9*Z*)/22:0) was the most abundant DAG species in pigs, donkeys and cattle. In sika deer, camels, goats and horses, DAG (P-14:0/18:1) predominated. DAG (20:0/20:4) was the most abundant DAG in the yak. MAGs were minor components in all lipid extracts, with MAG 18:1 and MAG 20:4 as the major species.

#### 3.2.4. ACar and MGDG Profiles

In the lipid extracts from the spinal cords of eight domesticated animals, ACar was analyzed in positive mode at levels ranging from 0.02% to 0.37% of the total lipids. ACar 18:1 was the predominant ACar species found in spinal cord lipid extracts from all animal species, except for goats. Concurrently, when analyzed in negative mode, MGDG comprised between 0.002% and 0.05% of the total lipids from the spinal cord extracts. It was noted that the fatty acyls at the *sn*-1 position of MGDG were mainly C16:0, C16:1 and C18:1, whereas the *sn*-2 position was primarily occupied by PUFAs.

The stability of the entire method is better when there is a higher correlation among QC samples (closer to 1). In this study, the square of Pearson’s correlation coefficient (R^2^) for QC samples ranged from 0.975 to 0.993 in positive mode and from 0.968 to 0.993 in negative mode, demonstrating excellent stability of the UHPLC-MS/MS-based metabolomics analysis. Additionally, the QC samples displayed tight clustering in the PCoA score plot, suggesting a high level of reliability of the metabolomics analysis. Further, throughout the entire analytical process, the QC samples introduced into the mass spectrometry system remained tightly clustered, emphasizing instrumental reproducibility and consistent stability throughout the experiment.

### 3.3. Correlation between Genomic Sequence and Lipidome

**Figure 4:**
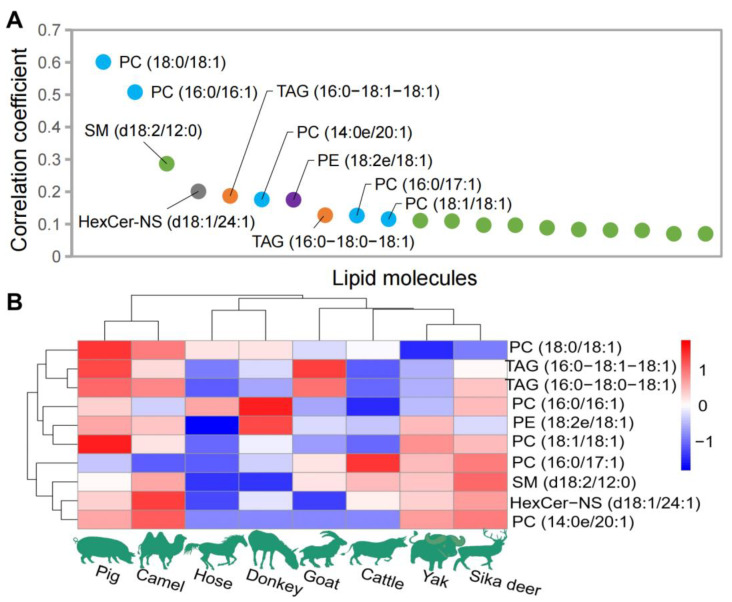
Lipid data analysis closely related to genomic data. (**A**) The top 20 lipids with higher weight coefficients. (**B**) Distribution of the top 10 lipids with the highest weight coefficient among the eight domesticated animals.

The correlations between genomic sequence data and lipidomic data obtained from eight species of domesticated animals were analyzed using the CCA algorithm As depicted in Figure 4, the lipids with the most significant correlations with the genomic sequence included PC [PC (18:0/18:1), PC (16:0/16:1), PC (14:0e/20:1), PC (16:0/17:1), PC (18:1/18:1), PC (16:0/22:6), and PC (17:0/18:1) and PC (14:0e/22:2)], PE [PE (18:2e/18:1), PE (16:1e/18:1), PE (18:2e/20:1), PE (18:0/18:1) and PE (P-18:0/18:2(9*Z*,12*Z*))], TAG [TAG (16:0/18:1/18:1), TAG (16:0/18:0/18:1), TAG (18:0/18:0/18:1) and TAG (18:0/18:1/18:1)], HexCer-NS [HexCer-NS (d18:1/24:1) and HexCer-NS (d18:1/24:0)] and SM [SM (d18:2/12:0)] (Figure 4A). Figure 4B shows the distribution of the top 10 lipids with the highest weight coefficient among the eight domesticated animals. Based on the clustering of the relative content of these 10 lipids, horses and donkeys still clustered together, pig and camel clustered together, and other animals formed a separate cluster.

## 4. Discussion

To the best of our understanding, this study is the first to provide a comprehensive lipidomic analysis of lipid molecular species in the spinal cords from various widely farmed livestock species. Our findings reveal a high degree of similarity in the lipid molecular species across the spinal cords of eight livestock species. However, we also observed significant variations in the content of each lipid class.

While comprehensive lipidomic data for mammalian spinal cords have not been documented, several studies have reported on the fatty acid compositions and lipid classes in the bone marrow. Notably, the early comprehensive studies of bovine lumbar bone marrows by Mello et al. [22] and Miller et al. [23] demonstrated that palmitic acid, stearic acid and oleic acid were the predominant fatty acid species. These findings align with the fatty acid profiling results from our study. Interestingly, our study revealed that the most abundant fatty acid species varied across the spinal cords of different domesticated animals. Moreover, Miller et al. identified TAG as the major lipid class in bovine lumbar bone marrows, with a content ranging from 100 to 160 g/100 g of fresh marrow [23]. PL, present at a level of 0.3–0.5 g/100 g of fresh marrow, was also identified in the lipid extracts from bovine lumbar bone marrows, including PC, PS, PG, DPG, PE, PA, PI, LPC and LPE. In contrast, our study found that PL was the predominant lipid class in spinal cords, followed by sphingolipids and glycerolipids. Morin et al. [24] investigated the FA composition of different bone marrows in caribou and found that palmitic acid and oleic acid were present at similar levels (~30%), while stearic acid was present at a level of ~27% in the lumbar marrow. Steiner-Bogdaszewska et al. reported an extremely low level (~3%) of stearic acid in the metatarsal bone marrows of red and fallow deer [25]. In comparison to Morin’s findings, our study found that, in the spinal cords of sika deer, oleic acid was present at a similar level. In contrast, palmitic and stearic acids were only at ~50% and ~65% of the reported levels, respectively. Furthermore, Cordain et al. (2002) reported on the fatty acid composition of rear metatarsal bone marrow in elk and deer [26]. In contrast to our findings in spinal cords, they reported a relatively low content of SAT (19~23%) and a relatively high content of MUFA (65~67%). Surprisingly, they also reported that palmitoleic acid was present at a significantly higher level (7–16%) than that we observed (<0.5%) in our study. In summary, this study provides a novel, comprehensive profiling of spinal cord lipid composition across eight livestock species. While some differences were noted between studies, overall similarities were found in major lipid species. Further research into lipidomic variations between central nervous system regions may reveal new insights into neurological disease mechanisms.

Morphologically, spinal cords are composed of white and gray matter. Imaging mass spectrometry enables the direct determination of spatial distributions of lipids in this nerve tissue. Previous studies using desorption electrospray ionization (DESI) mass spectrometry have reported that GPs and SLs are predominantly found in the white matter, while the gray matter exhibits a higher concentration of free fatty acids [27]. Moreover, it was reported that PC was identified in a slightly greater abundance than PE in gray matter. In contrast, PE was abundant more than PC in white matter [28,29,30]. More specifically, the SM species containing very long-chain fatty acids were found in high abundance in young wild-type C57BL/6 male mice [8], consistent with our findings in the spinal cords of domesticated animals. Furthermore, the correlation analysis of the lipidomic and genome data demonstrated the importance of PC, PE, TAG, HexCer-NS and SM. These lipid molecular species may play a significant role in the functioning of mammalian nerve tissues. Future studies should investigate the specific functions of these lipids in neuronal signaling and myelination. Our lipidomic dataset provides a valuable resource for comparative research on lipid composition in mammalian spinal cords.

## 5. Conclusions

In the currently study, we have utilized an untargeted lipidomic approach based on UPLC-Q-Exactive Orbitrap-MS to comprehensively investigate the lipid molecular species in the lipid extracts from the spinal cords of eight domesticated animal species. This study represents the first comprehensive lipidomic profiling of spinal cord tissues across multiple domesticated animal species. Through correlation analysis of genomic sequence data and lipidomic data based on the CCA algorithm, we have demonstrated the significant enrichment and importance of PC, PE, TAG, HexCer-NS and SM in the spinal cords. This research significantly advances our understanding of the contribution of each lipid molecular species to neurodevelopment. The comprehensive lipidomic analysis presented herein offers profound insights into the fat composition of spinal cords from domesticated animals. Furthermore, this study is anticipated to substantially enhance the high-value utilization of these byproducts in the meat-processing industry, thereby underscoring its practical implications.

## Figures and Tables

**Table 1:** Molecular species and content (% of total FAMEs) of FAs in spinal cords from eight species of domesticated animals.

FAs	Spinal Cords							
	Pig	Cattle	Yak	Goat	Horse	Donkey	Camel	Sika Deer
10:0	nd	nd	nd	0.10 ± 0.06	0.03 ± 0.01	nd	nd	nd
12:0	nd	nd	nd	0.22 ± 0.04	0.14 ± 0.03	nd	nd	nd
14:0	0.34 ± 0.03	0.23 ± 0.05	0.25 ± 0.07	2.85 ± 0.25	3.31 ± 0.51	0.52 ± 0.02	1.37 ± 0.03	0.39 ± 0.03
14:1 (n-5)	nd	nd	nd	0.06 ± 0.02	0.20 ± 0.03	nd	nd	nd
15:0	nd	nd	nd	0.25 ± 0.02	0.18 ± 0.01	nd	0.30 ± 0.007	0.17 ± 0.05
16:0	13.75 ± 0.80	11.67 ± 0.16	11.01 ± 2.83	20.29 ± 0.99	22.64 ± 1.71	9.48 ± 0.02	14.19 ± 0.76	15.55 ± 0.67
16:1 (n-7)	0.59 ± 0.03	0.25 ± 0.08	0.12 ± 0.05	0.87 ± 0.007	4.22 ± 0.49	0.36 ± 0.06	0.58 ± 0.06	0.49 ± 0.007
17:0	0.15 ± 0.06	0.83 ± 0.06	0.74 ± 0.16	0.46 ± 0.03	0.26 ± 0.002	0.12 ± 0.03	0.48 ± 0.04	0.58 ± 0.01
18:0	19.38 ± 1.53	21.24 ± 0.09	21.54 ± 2.68	16.39 ± 0.06	7.91 ± 0.52	22.69 ± 0.93	25.63 ± 0.41	18.49 ± 1.13
*trans* 18:1 (n-9)	0.22 ± 0.14	0.55 ± 0.17	0.90 ± 0.23	0.26 ± 0.08	0.26 ± 0.06	0.67 ± 0.21	0.93 ± 0.22	0.86 ± 0.23
*cis* 18:1 (n-9)	36.12 ± 2.87	36.07 ± 0.45	34.38 ± 2.65	36.04 ± 0.34	24.77 ± 0.61	28.98 ± 1.05	35.28 ± 0.31	29.56 ± 0.11
18:1 (n-7)	4.94 ± 0.12	2.89 ± 0.07	3.43 ± 0.71	1.73 ± 0.01	1.81 ± 0.11	4.04 ± 0.04	4.10 ± 0.06	4.72 ± 0.11
*cis* 18:2	4.51 ± 0.37	0.42 ± 0.13	0.10 ± 0.04	1.78 ± 0.14	21.84 ± 1.03	1.33 ± 0.16	0.42 ± 0.04	0.83 ± 0.02
18:3 (n-3)	0.17 ± 0.05	nd	nd	0.11 ± 0.007	2.41 ± 0.11	nd	nd	nd
20:0	1.11 ± 0.11	0.78 ± 0.04	0.64 ± 0.16	1.23 ± 0.10	0.50 ± 0.16	1.88 ± 0.08	0.36 ± 0.11	1.02 ± 0.28
20:1 (n-9)	4.65 ± 0.21	7.40 ± 0.11	9.21 ± 2.03	6.86 ± 0.29	2.97 ± 0.69	9.23 ± 0.23	3.02 ± 0.27	7.11 ± 0.11
20:3 (n-6)	0.56 ± 0.10	0.92 ± 0.25	0.88 ± 0.21	0.20 ± 0.007	0.59 ± 0.17	1.15 ± 0.29	0.97 ± 0.06	0.61 ± 0.13
20:4 (n-6)	2.24 ± 0.14	1.14 ± 0.07	1.27 ± 0.65	1.07 ± 0.06	0.52 ± 0.13	1.46 ± 0.23	0.91 ± 0.04	1.38 ± 0.04
22:0	2.05 ± 0.05	1.56 ± 0.44	1.24 ± 0.31	2.00 ± 0.04	0.27 ± 0.10	1.74 ± 0.08	0.61 ± 0.20	2.74 ± 0.54
22:1 (n-9)	0.76 ± 0.21	1.51 ± 0.13	1.15 ± 0.13	1.43 ± 0.22	0.50 ± 0.24	1.28 ± 0.23	0.56 ± 0.01	1.67 ± 0.04
23:0	0.40 ± 0.13	1.50 ± 0.27	0.85 ± 0.21	0.36 ± 0.05	0.26 ± 0.13	0.80 ± 0.29	0.48 ± 0.007	1.40 ± 0.21
24:0	2.54 ± 0.19	3.36 ± 0.48	3.06 ± 0.18	1.62 ± 0.15	1.53 ± 0.64	5.93 ± 0.22	2.02 ± 0.08	4.74 ± 0.45
24:1 (n-9)	5.52 ± 0.05	7.68 ± 0.45	9.23 ± 0.33	3.83 ± 0.09	2.88 ± 1.12	8.35 ± 0.13	7.82 ± 0.42	7.70 ± 0.02
∑ SFA	39.73 ± 1.69	41.17 ± 0.98	39.34 ± 2.82	45.77 ± 0.90	37.04 ± 0.73	43.15 ± 0.17	45.41 ± 1.24	45.06 ± 3.15
∑ MUFA	52.79 ± 2.74	56.34 ± 0.45	58.41 ± 3.27	51.07 ± 0.76	37.60 ± 0.85	52.91 ± 1.48	52.29 ± 0.49	52.11 ± 0.42
∑ PUFA	7.49 ± 0.73	2.49 ± 0.03	2.25 ± 0.33	3.16 ± 0.20	25.36 ± 0.85	3.94 ± 0.67	2.29 ± 0.06	2.82 ± 0.15

nd, not detected; SFA, saturated fatty acid; MUFA, monounsaturated fatty acid; PUFA, polyunsaturated fatty acid.

## Data Availability

The data used to support the findings of this study can be made available by the corresponding author upon request.

## Abbreviations

PC, Phosphatidylcholine; LPC, Lysophosphatidylcholine; PE, Phosphatidylethanolamine; LPE, Lysophosphatidylethanolamine; PS, Phosphatidylserine; LPS, Lysophosphatidylserine; PI, phosphatidylinositol; LPI, Lysophosphatidylinositol; PG, Phosphatidylglycerol; LPG, Lysophosphatidylglycerol; PA, phosphatidic acid; HBMP, Hemibismonoacylglycerophosphate; PEtOH, Phosphatidylethanol; ACar, Acyl-carnitine; SM, Sphingomyelin; Cer, Ceramide; GluCer, Glucosylceramide; SHexCer, Sulfatides hexosyl ceramide; MGDG, monogalactosyldiacylglycerol; MAG, Monoacylglycerol; DAG, Diacylglycerol; TAG, Triacylglycero; MeCN, Methyl cyanide.

## References

[1] Toldrá F., Mora L., Reig M. (2016). New insights into meat by-product utilization. Meat Sci..

[2] Palm W., Rodenfels J. (2020). Understanding the role of lipids and lipoproteins in development. Development.

[3] Sud M., Fahy E., Cotter D., Brown A., Dennis E.A., Glass C.K., Merrill A.H., Murphy R.C., Raetz C.R., Russell D.W. (2007). LMSD: LIPID MAPS structure database. Nucleic Acids Res..

[4] Harayama T., Riezman H. (2018). Understanding the diversity of membrane lipid composition. Nat. Rev. Mol. Cell Biol..

[5] Sunshine H., Iruela-Arispe M.L. (2017). Membrane lipids and cell signaling. Curr. Opin. Lipidol..

[6] Lin Z., Wu Z., Huang C., Lin H., Zhang M., Chen M., Han K., Huang W., Ruan S. (2023). Cloning and expression characterization of elongation of very long-chain fatty acids protein 6 (elovl6) with dietary fatty acids, ambient salinity and starvation stress in *Scylla paramamosain*. Front Physiol..

[7] Yoon J.H., Seo Y., Jo Y.S., Lee S., Cho E., Cazenave-Gassiot A., Shin Y.S., Moon M.H., An H.J., Wenk M.R. (2022). Brain lipidomics: From functional landscape to clinical significance. Sci. Adv..

[8] Wang C., Palavicini J.P., Han X. (2021). A Lipidomics Atlas of Selected Sphingolipids in Multiple Mouse Nervous System Regions. Int. J. Mol. Sci..

[9] Chaves-Filho A.B., Pinto I.F.D., Dantas L.S., Xavier A.M., Inague A., Faria R.L., Medeiros M.H.G., Glezer I., Yoshinaga M.Y., Miyamoto S. (2019). Alterations in lipid metabolism of spinal cord linked to amyotrophic lateral sclerosis. Sci. Rep..

[10] Zhai J., Ström A.L., Kilty R., Venkatakrishnan P., White J., Everson W.V., Smart E.J., Zhu H. (2009). Proteomic characterization of lipid raft proteins in amyotrophic lateral sclerosis mouse spinal cord. FEBS J..

[11] Yang K., Zhao Z., Gross R.W., Han X. (2009). Systematic analysis of choline-containing phospholipids using multi-dimensional mass spectrometry-based shotgun lipidomics. J. Chromatogr. B Analyt. Technol. Biomed. Life Sci..

[12] Hou B., Zhao Y., He P., Xu C., Ma P., Lam S.M., Li B., Gil V., Shui G., Qiang G. (2020). Targeted lipidomics and transcriptomics profiling reveal the heterogeneity of visceral and subcutaneous white adipose tissue. Life Sci..

[13] Zhang H., Gao Y., Sun J., Fan S., Yao X., Ran X., Zheng C., Huang M., Bi H. (2017). Optimization of lipid extraction and analytical protocols for UHPLC-ESI-HRMS-based lipidomic analysis of adherent mammalian cancer cells. Anal. Bioanal. Chem..

[14] Xuan Q., Hu C., Yu D., Wang L., Zhou Y., Zhao X., Li Q., Hou X., Xu G. (2018). Development of a high coverage pseudotargeted lipidomics method based on ultra-high performance liquid chromatography-mass spectrometry. Anal. Chem..

[15] Yuenyong J., Pokkanta P., Phuangsaijai N., Kittiwachana S., Mahatheeranont S., Sookwong P. (2021). GC-MS and HPLC-DAD analysis of fatty acid profile and functional phytochemicals in fifty cold-pressed plant oils in Thailand. Heliyon.

[16] Matyash V., Liebisch G., Kurzchalia T.V., Shevchenko A., Schwudke D. (2008). Lipid extraction by methyl-tert-butyl ether for high-throughput lipidomics. J. Lipid Res..

[17] Fu X., Calderón C., Harm T., Gawaz M., Lämmerhofer M. (2022). Advanced unified monophasic lipid extraction protocol with wide coverage on the polarity scale optimized for large-scale untargeted clinical lipidomics analysis of platelets. Anal. Chim. Acta..

[18] Gangadhara R.M., Gowda S.G.B., Gowda D., Inui K., Hui S.P. (2023). Lipid Composition Analysis and Characterization of Acyl Sterol Glycosides in Adzuki and Soybean Cultivars by Non-Targeted LC-MS. Foods.

[19] Qadar M.A., Seghouane A.K. (2019). A Projection CCA Method for Effective fMRI Data Analysis. IEEE Trans. Biomed. Eng..

[20] Butler A., Hoffman P., Smibert P., Papalexi E., Satija R. (2018). Integrating single-cell transcriptomic data across different conditions, technologies, and species. Nat. Biotechnol..

[21] Li H., Kang X., Wang S., Mo H., Xu D., Zhou W., Hu L. (2021). Early detection and monitoring for aspergillus flavus contamination in maize kernels. Food Control.

[22] Mello F.C., Field R.A., Forenza S., Kunsman J.E. (1976). Lipid characterization of bovine bone marrow. J. Food Sci..

[23] Miller G.J., Frey M.R., Kunsman J.E., Field R.A. (1982). Bovine bone marrow lipids. J. Food Sci..

[24] Morin E., Soppela P., Chouinard P.Y. (2022). Thermal adaptation and fatty acid profiles of bone marrow and muscles in mammals: Implications of a study of caribou (*Rangifer tarandus caribou*). PLoS ONE.

[25] Steiner-Bogdaszewska Ż., Tajchman K., Domaradzki P., Florek M. (2022). Composition and Fatty Acid Profile of Bone Marrow in Farmed Fallow Deer (*Dama dama*) Depending on Diet. Animals.

[26] Cordain L., Watkins B.A., Florant G.L., Kelher M., Rogers L., Li Y. (2002). Fatty acid analysis of wild ruminant tissues: Evolutionary implications for reducing diet-related chronic disease. Eur. J. Clin. Nutr..

[27] Girod M., Shi Y., Cheng J.X., Cooks R.G. (2010). Desorption electrospray ionization imaging mass spectrometry of lipids in rat spinal cord. J. Am. Soc. Mass. Spectrom..

[28] Kim H.Y., Huang B.X., Spector A.A. (2014). Phosphatidylserine in the brain: Metabolism and function. Prog. Lipid Res..

[29] Söderberg M., Edlund C., Kristensson K., Dallner G. (1991). Fatty acid composition of brain phospholipids in aging and in Alzheimer’s disease. Lipids.

[30] Landgraf R.R., Prieto Conaway M.C., Garrett T.J., Stacpoole P.W., Yost R.A. (2009). Imaging of lipids in spinal cord using intermediate pressure matrix-assisted laser desorption-linear ion trap/Orbitrap MS. Anal. Chem..

